# HIV-1 Reverse Transcriptase/Integrase Dual Inhibitors: A Review of Recent Advances and Structure-activity Relationship Studies

**DOI:** 10.22037/ijpr.2021.115446.15370

**Published:** 2021

**Authors:** Mohammad Mahboubi-Rabbani, Maryam Abbasi, Zahra Hajimahdi, Afshin Zarghi

**Affiliations:** a *Department of Pharmaceutical Chemistry, School of Pharmacy, Shahid Beheshti University of Medical Sciences, Tehran, Iran.*; b *Department of Medicinal Chemistry, Faculty of Pharmacy, Hormozgan University of Medical Sciences, Bandar Abbas, Iran.*

**Keywords:** HIV-1, Reverse Transcriptase, Integrase, Dual Inhibitors, SAR

## Abstract

The significant threat to humanity is HIV infection, and it is uncertain whether a definitive treatment or a safe HIV vaccine is. HIV-1 is continually evolving and resistant to commonly used HIV-resistant medications, presenting significant obstacles to HIV infection management. The drug resistance adds to the need for new anti-HIV drugs; it chooses ingenious approaches to fight the emerging virus. Highly Active Antiretroviral Therapy (HAART), a multi-target approach for specific therapies, has proved effective in AIDS treatment. Therefore, it is a dynamic system with high prescription tension, increased risk of medication reactions, and adverse effects, leading to poor compliance with patients. In the HIV-1 lifecycle, two critical enzymes with high structural and functional analogies are reverse transcriptase (RT) and integrase (IN), which can be interpreted as druggable targets for modern dual-purpose inhibitors. Designed multifunctional ligand (DML) is a new technique that recruited many targets to be achieved by one chemical individual. A single chemical entity that acts for multiple purposes can be much more successful than a complex multidrug program. The production of these multifunctional ligands as antiretroviral drugs is valued with the advantage that the viral-replication process may end in two or more phases. This analysis will discuss the RT-IN dual-inhibitory scaffolds’ developments documented so far.

## Introduction

**Figure 1:**
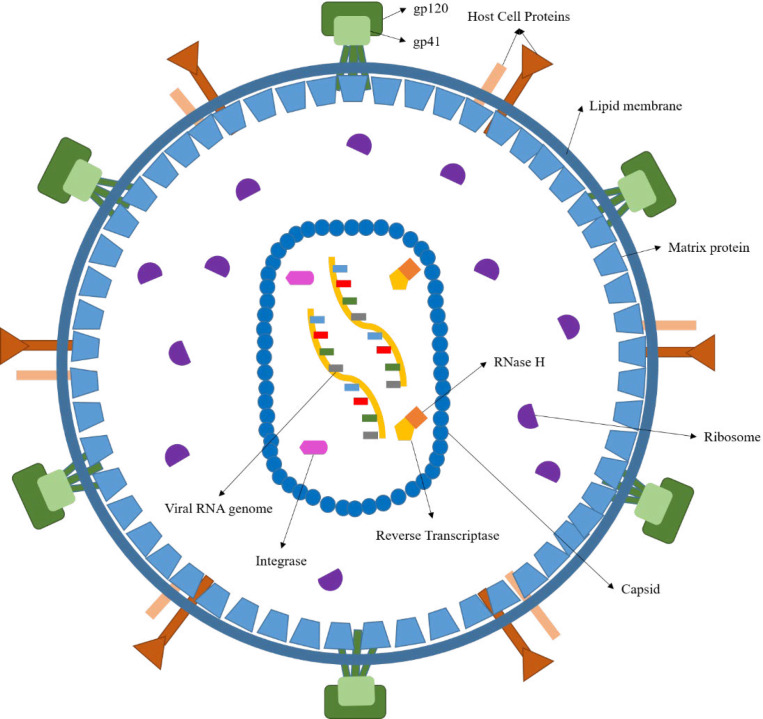
HIV virion structure

**Figure 2:**
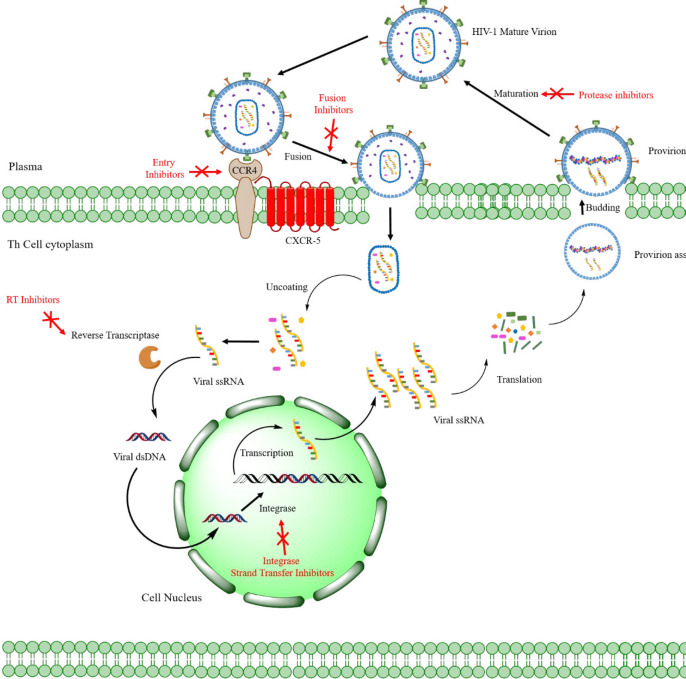
HIV-1 life cycle

Acquired Immunodeficiency Syndrome (AIDS) is a complex illness caused by the human immunodeficiency virus (HIV) (Figure 1) (1). More than 37 million people have recently been confirmed to be in an immunocompromised condition due to HIV infection. There are annual estimates of around 1.8 million new cases, and about 770,000 people worldwide died in 2018 from AIDS-related diseases (2). While a significant reduction in mortality has been achieved through the highly active antiretroviral therapy (HAART), HIV infection can only be temporarily managed but not eradicated (3-4). Furthermore, the HAART efficacy has been limited by the emergence of drug-resistant viral strains (5-6). As a result, developing new anti-HIV agents remains a crucial scientific initiative in medicinal research (7). HIV has been categorized into HIV-1 & HIV-2, the first of which, HIV-1, is most widespread and more infectious than HIV-2 (8). Considering the HIV-1 life cycle (Figure 2), reverse transcriptase (9) (RT), RT-dependent ribonuclease H (10-11) (RNase H), and integrase (10) (IN) play pivotal roles in stable infection, and it is understandable how these enzymes are attractive therapeutic targets for drug design. Currently, only drugs targeting the RT polymerase and IN strand transfer activity have been approved for clinical use, and RNase H inhibitors have received little consideration (11). However, RNase H’s catalytic site is very similar to that of IN, and many compounds can be found that may act as potent inhibitors for both enzymes (12-14). The importance of designing such dual-acting enzyme inhibitors has become increasingly apparent, especially with the rise of the designed multiple ligands (DML) concept in medicinal chemistry as a way to increase drug efficiency and reduce adverse drug reactions (ADR) (15-19). We addressed in our previous works the idea of dual targeting for some biological targets, including carbonic anhydrase/COX-2 and lipoxygenase/COX-2 dual inhibitors as cancer chemopreventives (20-22). Furthermore, we developed a dozen of novel compounds as IN inhibitors in recent years (23-30), in which some scaffolds were determined as promising lead compounds for further studies. However, their effectiveness as dual RNase H/IN inhibitors has been remained to be investigated either *in-vitro* or *in-vivo*. Herein, we briefly describe the scientific background of dual RT-associated RNase H/IN inhibitors, review the present status, and discuss further options in the field.

**Structure and replication cycle of HIV-1**

*HIV-1 structure & life cycle*

HIV is a round-shaped enveloped virus, measuring 100 nm in diameter, classified into the Retroviridae family within the Lentivirus class (31). 

The HIV genome comprises two identical single-stranded RNA (ssRNA) molecules enclosed in the virus’ capsid core (32) (Figure 1). As shown in Figure 2, the HIV life cycle commences by the attachment of virus-encoded glycoprotein 120 (gp120) to the CD_4_ receptors of helper T lymphocytes (T_h_). A conformational change in the gp120 then exposes the smaller gp-41 viral glycoprotein, which binds to the chemokine co-receptors, leading to the fusion of viral envelope and host cell membrane releasing viral material into the host cytoplasm (33). Henceforth, the HIV-1 genome will be created by reverse transcription of viral RNA into DNA, followed by incorporating HIV-1 double-stranded DNA (dsDNA) into the host cell genome by viral IN (34). 

In the viral DNA, the coding region of three main enzymes (RT, RNase H, and IN), known as the pol reading frame, contributes to the viral maturity of HIV in the new host cells (31) (Figure 2). While all of these may act as exploitable pharmaceutical targets, RT and IN are the foci for discovering and manufacturing dual-acting inhibitors. As discussed earlier, there is a highly streamlined, multi-target oriented therapy called DML, in which a single chemical scaffold confers activities against multiple therapeutically viable targets (35). In the continuation of this article, our focus will be on the above two enzymes ,and their dual inhibitors.

*The function of HIV-1 RT polymerase and RNase H*

The reverse transcription of the viral ssRNA into dsDNA is caused by the HIV-1 reverse transcriptase (34). RT consists of two functional areas; an active site with DNA polymerase and a dynamic site with RNase H (12). The polymerase’s active site is an ssRNA or ssDNA model, and the RNase H active site cleaves the RNA/DNA hybrid strand (36). In addition to viral IN incorporated in the host genome, both essential enzyme functions of RT work to transform the virus ssRNA into dsDNA (36-37). Most RT inhibitors (RTIs) introduced so far inhibit the polymerase function of the enzyme. In contrast, none of the approved antiviral agents, whether nucleoside reverse transcriptase inhibitors (NRTIs) or non-nucleoside ones (NNRTIs) inhibit specifically the necessary RNase H activity (12, 38). 

*The function of IN*

As first described by Mouscadet and Tchertanov (39-40), the integration of a DNA copy of the viral RNA genome into host chromatin is catalyzed by the virus-encoded IN, conserved among retroviruses (41). It may happen at any location in the host genome, but it is preferable for specific segments of DNA (42). Integrated viral DNA survives in the long-lived cell and is replicated through the sequence of cell divisions in conjunction with host DNA (43). This provides the latent reserve for high-viral rebounds as soon as the therapy stops or conditions are favorable (44). Reverse transcribed viral DNA, called the preintegration complex (PIC), is brought into the host cell nucleus in a nucleo-protein portion, incorporated into the host DNA (45). PIC comprises a few viral core and host proteins, in addition to the IN tetramer and viral DNA (46). The first stage of the integration process occurs in the cytoplasm, where IN removes two terminal nucleotides (G, T) from 3´-ends of the long terminal repeat (LTR) region of reverse-transcribed viral DNA as part of the PIC (47). This step concerns 3´-processing and involves phosphodiester bond hydrolysis (48). In a process that is called strand transfer, PIC translocates to the infected cell nucleus where the IN links to DNA, and terminal 3´-OH of the viral DNA attacks the host DNA (49). The viral and host strands are then totally sealed by nucleic acid repair enzymes following the ST process (50). As integration is a critical and unique milestone in the HIV-1 replication cycle, IN is considered an exciting drug target for HIV inhibitors (23). 

**Structural dynamics of selected targets**

*Structure of HIV-1 RT*

**Figure 3:**
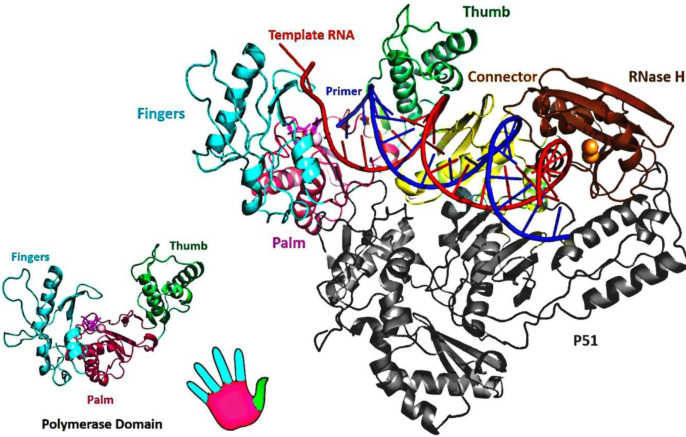
X-ray crystallographic structure of HIV-1 RT (PDB ID: 1RTD). The enzyme is crystallized bound with the nucleic acid template (red) and primer (blue).

HIV-1 RT consists of an asymmetrical heterodimer of 1000 amino acids composed of two associated structural subunits, p66 and p51 (51). P66 contains 560 amino acids, and 440 amino acids are found in smaller p51 (52). While p51 plays only structural function, the larger p66 domain has spatially separate active sites, including N-terminal polymerase (residues 1-318), connectivity (residues 319-426), C-terminal RNase H, as are shown in Figure 3 (53). The polymerase is described as resembling a right hand with three sub-domains: fingers (residues 1-85 and 118-155), palm (residues 86-117 and 156-236), and thumb (residues 237-318) (53). The binding region for DNA nucleic acids is primarily found within p66 and its sub-domains (fingers, palm, thumb, connector, and RNase H) (12). The floor of the nucleic acids binding area consists of the p51 connection and thumb subdomains (54). The binder site is folded to permit nucleic acid to reach polymerase and RNase H active sites; approximately 17-18 base pairs of the nucleic acid substrate are separated from each other (with a distance of ~60 Å) (54-56). 

**Figure 4:**
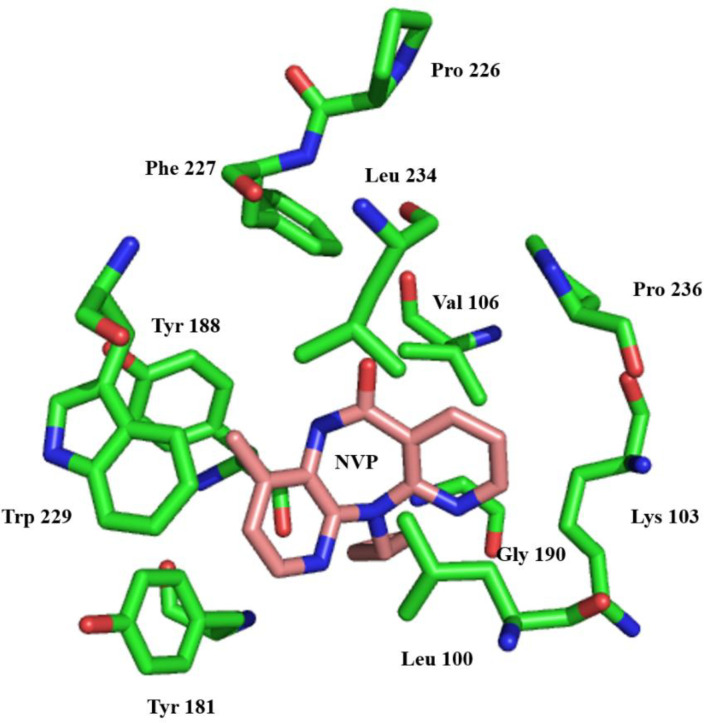
Stereo representation of the NNRTI-binding site of RT, showing the most significant protein-drug interactions. Nevirapine is shown in pink

Non-nucleoside reverse transcriptase inhibitor binding pocket (NNRTIBP) is an allosteric, hydrophobic pocket (~ 10 Å) near the polymerase’s active site, which lies primarily in the p66 region of the polymerase active site (57). It comprises several aromatic (Tyr-181, Tyr-188, Phe-227, Trp-229 & Tyr-232), hydrophilic (Lys-101, Lys-103, Ser-105, Asp-132, and Glu-224), and hydrophobic residues (Pro-59, Leu-100, Val-106, Val-179, and Leu-236) and two more amino acids (Ile-135 and Glu-138) from p51 subunits (58). In this binding pocket are all mutations causing NNRTI resistance (59) (Figure 4).

*Polymerase active site of HIV-1 RT*

**Figure 5:**
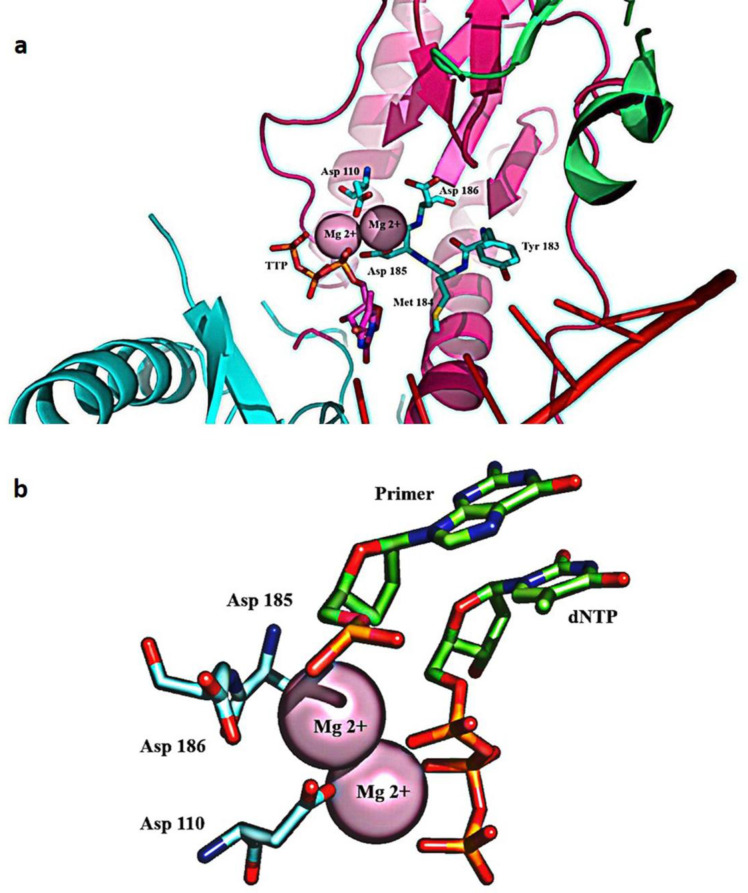
(a) The polymerase active site comprises two magnesium coordinating catalytic aspartate residues in the palm subdomain of p66. D185 and D186 are part of the YXDD motif, highly conserved in retroviral RTs; (b) Magnesium coordination at the polymerase active site of RT

The palm subdomain contains three catalytic carboxylates (Asp110, Asp185, and Asp186), which bind two Mg^2+^ divalent ions needed for catalytic processing (60). In retroviral systems (with X=Met in HIV-1 RT), Asp 185 and Asp186 are part of the Y (X) DD motif, which is preserved. Mg^2+^ ions coordinate with the oxygen atoms of three phosphates of the inbound dNTP and the three catalytic Asp residues during the polymerization reaction, facilitating the primer 3-OH nucleophilic attack on the α-phosphate of the incoming dNTP (61) (Figure 5). 

*Structure of HIV-1 RNase H*

HIV-1 RNase H was the first segment of *RT* to be crystallized (12). Viruses lacking RNase H function are non-infectious, making RNase H a fascinating target for anti-HIV therapeutics (62). HIV-1 RNase H is structurally similar to other forms of ribonucleases, such as *Escherichia coli (E. coli)* and *Thermus thermophilus* *(T.* *thermophilus)* (63). The RNase H active site’s crystal structure shows two Mg^2+^ at a distance of ~4 Å from each other, forming a coordination complex with highly conserved *Asp442*, *Glu478*, *Asp498*, and *Asp549* residues (64). Studies on RNase H confirmed a mechanism dependent on two metal ions for substrate recognition and catalysis that is functionally similar to other retroviral integrases (36).

*Structure of HIV-1 IN*

**Figure 6:**
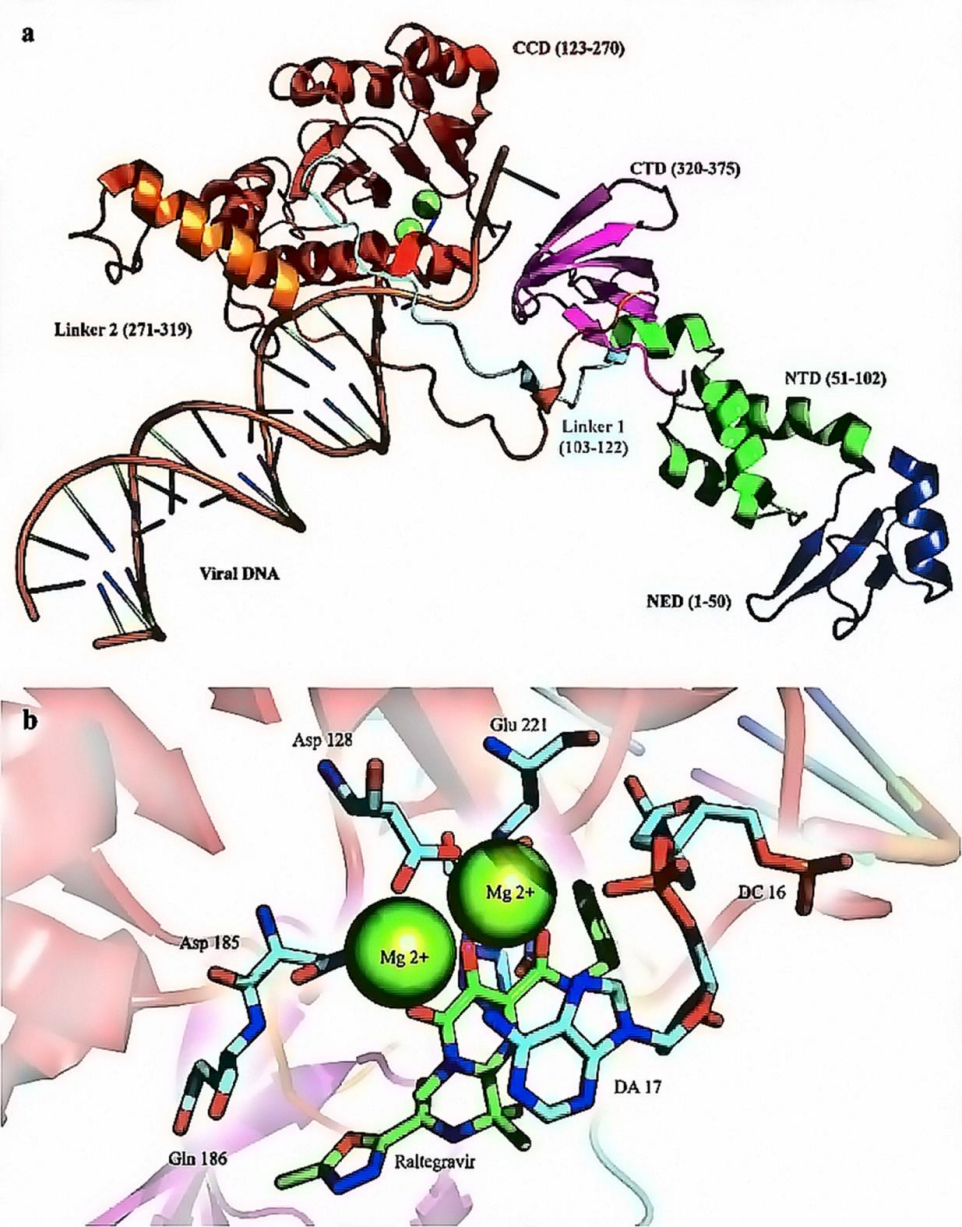
(a) X-ray crystallographic structure of HIV-1 IN (PDB ID: 3OYA). The enzyme is crystallized bound with the nucleic acid template (orange); (b) The IN active site comprises three magnesium coordinating catalytic residues in the palm subdomain, D128, D185, and E221

As a member of the poly-nucleotidyl transferases superfamily, *IN* is a multi-subunit 32 kDa protein, possessing 288 amino acids (65). IN plays a dual role with its dimeric configuration, catalyzing the 3´-P step and tetrameric arrangement, which transfers nucleic acid strands in ST phase (62). Each IN monomer comprises three distinct structural domains, N-terminal, C-terminal, and core catalytic domains (66). N-terminal contains an HHCC zinc finger with four conserved residues (His12, His16, Cys40, and Cys43) required for enzyme multimerization (67). The catalytic core domain (CCD), which comprises five β-sheet strands surrounded by six α-helices linked together by flexible loops, contains two Mg^2+^ ions in coordination with Asp64, Asp116, and Glu152 residues (68). The least conserved C-terminal domain comprising amino acids 213-288 binds nonspecifically with the DNA, which its linkage with CCD is essential for 3´-P & ST activities (68-69). IN flexibility allows it to interact with numerous proteins to perform a multifunctional role in viral replication (46, 70). The crystallographic characterization of IN largely remains elusive except for *prototype foamy virus* (*PFV*) integrase, which has an extended N-terminal domain and longer linkers, only shares limited sequence similarity with HIV-1 IN (69) (Figure 6). Nevertheless, the recently-characterized molecular structure of the HIV-1 IN-DNA complex (intasome) has proven that the HIV-1 and *PFV* intasomes are fairly identical (71). Therefore, studying the structure of the dolutegravir *PFV* intasome complex may help elucidate the mechanism of IN inhibition by INSTIs.

*IN and RNase H: What is similar, and what is different?*

**Figure 7:**
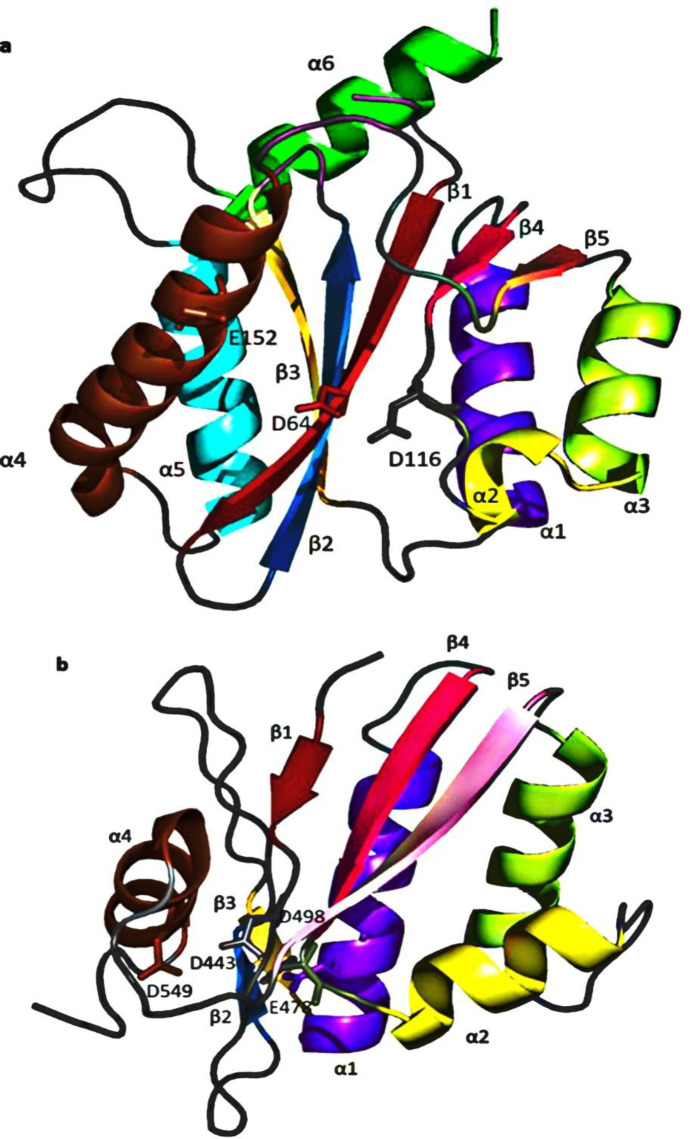
Comparison of the (a) HIV-1 IN (PDB code: 1BIS) and (b) RNase H (PDB code: 1R0A) tertiary structures, showing corresponding β-sheet and α-helices labeled as follows (β_1_, red; β_2_, sky blue; β_3_, yellow-orange; α_1_, purple-blue; β_4_, hot pink; α_2_, yellow; α_3_, lemon; β_5_, pink; α_4_, brown; α_5_, cyan; α_6_, green. Amino acids of the DDE motif forming the catalytic site of both enzymes are labeled in white

IN and RNase H’s active sites are composed of five-stranded β sheets surrounded by α helices with the same metal-coordinating DDE amino acid triad essential for catalytic activity. Additionally, both use similar divalent metal cofactors during their catalytic activity (72-73). The five strands of the IN β sheets superimpose closely with the corresponding RNase H sheets (Figure 7). However, some specific properties were found; for instance, IN α_1_ is displaced from the four β sheets by approximately 6 Å relative to the RNase H α_1_. Moreover, IN α_2_ is a one-turn helix, while RNase H α_2_ is a three-turn helix. Two additional helices are found in IN that is not present in RNase H (74). IN α_5 _(residues 171-186) is located adjacent and parallel to β_3_, whereas α_6_ (residues 196-208), located on the same side as α_1_, with an orientation of approximately 90° to the α_5_. With all this, the reasonably overlapping pharmacophores for both enzymes rationalize their exploitation as potential targets for developing dual inhibitors (75). By contrast, the RNase H site possesses four highly conserved amino acid residues, including D443, E478, D498, and D549 (Figure 7), which entirely neutralize the Mg^2+^ ions making the pocket electrostatically neutral (76).

On the other hand, in the IN active site, only three acidic residues (D64, D116, and E152) oppose the positive charges mounted by the Mg^2+^ ions. This allows the negatively charged carboxylate moiety in the acidic molecules to bind more favorably with HIV-1 IN than RNase H (77). 

Interestingly, both IN and RNase H inhibitors share a hydrophobic side chain that binds with the enzyme active sites’ corresponding residues. However, the orientation of these side groups is different from each other in a manner that helps to design more specific enzyme inhibitors.

**Drugs acting on selected targets**

*HIV-1 RT inhibitors*

**Figure 8:**
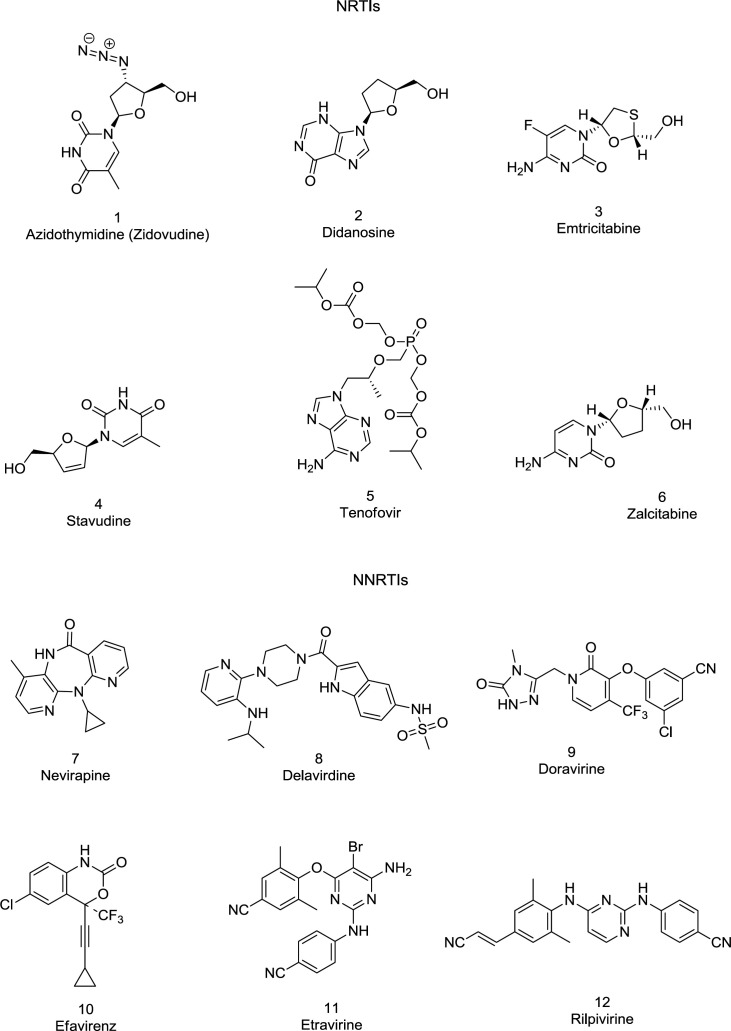
FDA-approved NRTIs and NNRTIs

Approximately half of the anti-HIV drugs target the RT polymerase activity. All approved anti-RT drugs belong to one of two main classes: the nucleoside/nucleotide RT inhibitors (NRTIs/NtRTIs) bind specifically with the enzyme catalytic site, whereas the NNRTIs are non-competitive inhibitors, binding with an allosteric site, named the NNRTIBP located within the primer grip region and Tyr181/Tyr188 aromatic rings (78). Some FDA-approved RT (**1-12**) inhibitors are shown in Figure 8. 

Structural studies suggest that NNRTIs binding alters the catalytic carboxylate triad (Asp110, Asp185, and Asp186) that binds the Mg^2+^ ions, enforcing the conserved YMDD motif to assume an inappropriate conformation during DNA synthesis (79).

*HIV-1 RNase H inhibitors*

**Figure 9:**
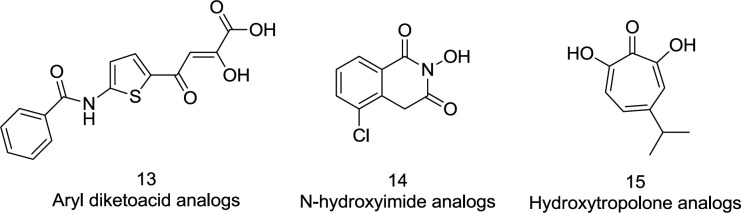
RNase H active site inhibitors

RNase H is a worthy and promising goal to develop new antiretroviral drugs. Nevertheless, RNase H inhibitors have slowed development, and no success story has yet been published. It may be due to two causes: the extraordinarily complex and costly *in-vitro* RNase H research and human equivalents close to RT RNase H necessary for the host’s survival cells (11). Over the past decade, however, the role of RNase H has been re-examined, and several groups of RNase H inhibitors, including aryl diketo acid (DKA) derivatives (**13**) and other scaffolds such as N-hydroxy imide (**14**) and hydroxy tropolone (**15**), have been identified (44) (Figure 9). Ironically, the diketo acid class was well distinguished also for its inhibitory role of IN.

*HIV-1 IN ST inhibitors*

**Figure 10:**
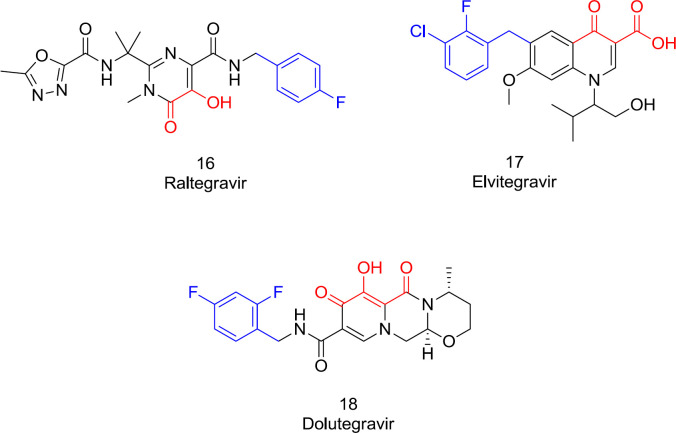
HIV-1 IN inhibitors with the metal coordinating motif (red) and the hydrophobic group (blue).

Drugs that target IN have been developed and successfully used, combined with proven RT and PR inhibitors (80). Through the integration process, there are three biological targets to drug design: inhibition of preintegration complex (PIC) assembly, inhibition of 3’-end processing (3’-P), and strand transfer (ST) inhibition (81). Two primary IN inhibitor classes include protein-protein interaction inhibitors (PPIs) and integrase ST inhibitors (INSTI) (49). The INSTIs target the IN active site preventing the PIC from binding to the host genome. All currently-available FDA-approved IN inhibitors are INSTIs (Figure 10) (49). INSTIs have a low affinity for IN alone but show a high affinity for the intasome (IN-DNA complex). In combination with the viral DNA, the enzyme produces a conformational modification needed for high-affinity drug interaction (46). Two structural requirements for the IN binding scaffold are essential: a hydrophobic motif anchored in a highly hydrophobic region near the catalytic area; and a metal-coordinating triad, chelating two metal ions within a hydrophilic pocket (82). Over the past years, our research team expanded the metal coordinating pharmacophore’s scope to explore potential integrase inhibitors of HIV-1 IN (23-26).

The first approved INSTIs for clinical use, including raltegravir (**16**), elvitegravir (**17**), and dolutegravir (**18**), developed viewing these pharmacophoric requirements, sharing these two standard chemical features needed for the IN inhibitory activity (Figure 10). Indeed, all the potent INSTIs possess the hydrophobic mostly in the form of a substituted benzyl that inhibits the ST phase by displacing the 3’-end adenine on the viral DNA to a misaligned conformation (62). This hydrophobic motif should be in a planar position relative to the metal-coordinating group for maximum strength. Some studies have shown that by inserting a meta-halide, the benzyl motif interaction with the IN hydrophobic pocket and some other structurally-related enzymes like Hepatitis C virus NS5B will be further improved (83-84).

**Multiple-acting Ligands**

*Classification of RT-associated RNase H/IN dual inhibitors*

As described before, the inhibition mechanism for both INST and RT RNase H is crucially dependent on the metal-chelating propensity of the enzyme inhibitors (85). Hence, while a few chemical nuclei have been explored for their dual inhibitory effects against HIV-1 RT and IN, a vast number of RT/IN dual inhibitors have been introduced in the literature if the similarities between both enzymes are considered. Following are a few promising classes of RT/IN inhibitors. 

**DKAs**

*Diketoacid analogs*

**Figure 11:**
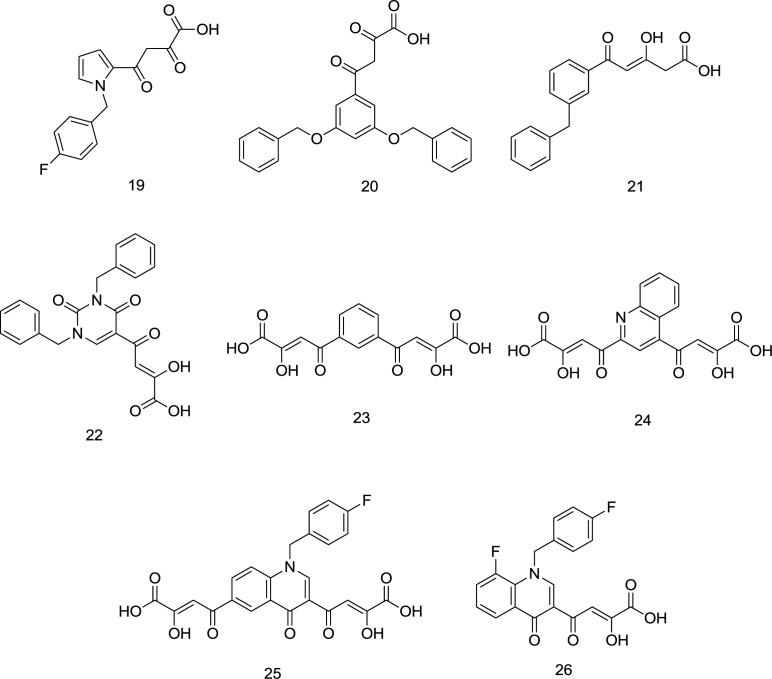
DKA analogs

**Figure 12:**
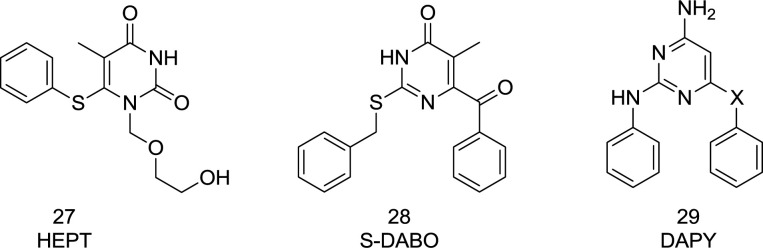
S-DABO, HEPT, and DAPY

One of the oldest researches concerning the design and synthesis of IN inhibitors dates back to 2000, in which a series of aryl diketo acid analogs, exemplified by the molecules **19-20,** were developed by Huzada and coworkers (86). Two pharmacophores are reported to find in the typical DKAs as IN inhibitors: an aromatic motif bonded to a hydrophobic cavity near the active site of the IN; and a pharmacophoric DKA motif involved in the metal coordination to create a triad complex between the ligand, IN and metal ions that interferes with the IN activity (87). It was discovered that the closer the angle between the chelator group and the hydrophobic ring to 120° (compound **20**), the higher the inhibitory effect on the IN enzyme. Moreover, ortho substitution of the distal benzyl ring with halogens was found to enhance the INST inhibitory activity, while the para-substitution leads to an approximately inactive compound. In another study conducted by Wai and colleagues, analogous INST inhibitors were developed through the central benzene ring alkoxylation in the compound **20** (88) (Figure 11). Analogs like compound **22 **(Figure 11) demonstrated striking structural analogy to hydroxyl ethoxy phenylthio thymine (HEPT) (Figure 12), S-dihydroalkoxy benzyl-oxopyrimidine derivatives (S-DABOs) (Figure 12), the diaryl aminopyrimidine (DAPY) (Figure 12), and NNRTIs like etravirine (Figure 8) (89). The findings mentioned above were followed up by hybridizing these compounds and DKAs moiety to create potent RT/IN dual inhibitors. In 2006, Di Santo *et al.* introduced a series of bifunctional DKA INSTIs, including compounds **22-24 **(Figure 11), indicating less potent antiviral activity than mono-functional DKA derivatives with high activity against both the 3’-P and ST phases (90). 

As shown in Figure 11, these compounds lacked the essential hydrophobic moiety except for compound **22**, which was the most potent IN inhibitor in the series. In continuation, four new series of mono-functional diketo acid (MDKA) derivatives of **22** were synthesized by the same research group as selective inhibitors of INST function in which compound **23 **(Figure 11) was the most active (91). Based on the resemblance between RNase H and IN, Di Santo *et al.* evaluated some pyrrolyl and quinolonyl DKA analogs for their dual inhibitory action against both the enzymes and found promising results (92). It was then that more studies were conducted on the compounds of these two families to introduce dual RNase H/IN inhibitors.

*Pyrrolyl DKA analogs*

**Figure 13:**
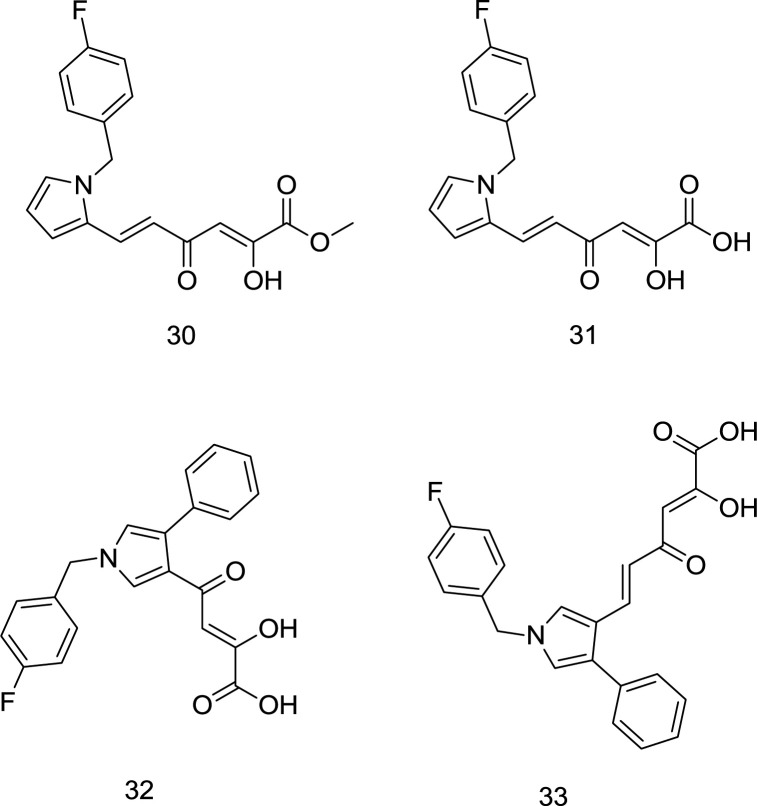
Pyrrolyl DKA derivatives

As mentioned earlier, a wide variety of host ribonucleases, which resembles RT RNase H and is also pivotal for the host cell’s proper functioning, is one of the key reasons why RNase H is not vastly exploited as a target for drug design. Interestingly, Tramontano *et al.* identified a pyrrolyl DKA analog **30 **(Figure 13) as a potent RT RNase H inhibitor with no activity against the DNA polymerase function of RT (93). 

In 2013, Costi and coworkers introduced a novel series of pyrrolyl DKA analogs as RT-associated RNase H/IN dual inhibitors in which compound **31** (Figure 13) was identified as the most potent inhibitor with IC_50_ values of 2.5 μM and 26 nM, for RNase H and IN enzymes, respectively (94). Interestingly, esterified analogs of compound **31** demonstrated a little higher degree of selectivity for HIV-1 RNase H versus IN, while acid derivatives were more active against IN. Indeed, ester functionality was more appropriate for dual targeting of IN and RNase H, perhaps due to the different electrostatic properties of the two enzymes’ active sites elaborated earlier. The findings mentioned above were followed up by performing SAR analysis (95). In summary, the best inhibitory activity was acquired when the DKA chain and a phenyl moiety were placed on positions 3 and 4 of the pyrrole ring, respectively, as observed in compounds **32** and **33** (Figure 13). Moreover, SAR analysis demonstrated that the replacement of terminal COOH with triazolyl reduced inhibitory activity against both enzymes.

*Pyridin-2-one DKAs analogs*

**Figure 14:**
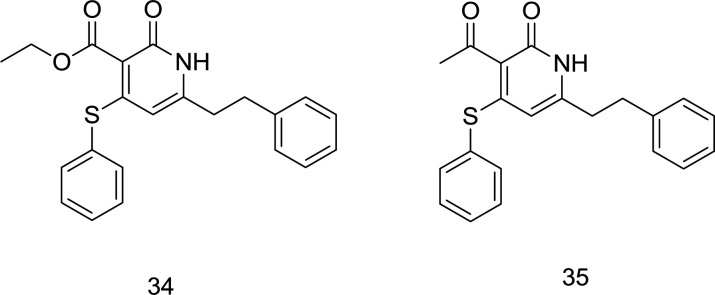
Pyridine DKA analogs

In 2017, three series of novel dual RT/IN inhibitors, including sulfide, sulfoxide, and sulfone analogs, were rationally designed by attaching a DKA motif into the pyridin-2-one scaffold, in which the molecules bearing ester or acetyl groups on C_3_ position were shown to possess an enhanced binding affinity (96). It is noteworthy that N-benzyl carbamoyl possessed a motivating ability to improve binding affinity compared to other amines. Due to the steric hindrance caused by the oxygen atoms in the C_4_ position, which made it unfavorable for the molecule to interact with NNRTIs binding pocket, the sulfide series showed a better dual HIV-1 RT/IN inhibitory activity than both sulfoxide and sulfone series. Therefore, the C_4 _substituent’s steric effect was speculated to regulate the conformation of the C_3_ group, which may further affect inhibitory activity against both enzymes. From the biological results, the compounds **34** and **35** (Figure 14) were identified as the most potent dually-acting RT/IN inhibitors of this series; however, the latter possessed more inhibitory activity against IN with an inhibition rate of 49.5%.

*Quinoline DKA analogs*

**Figure 15:**
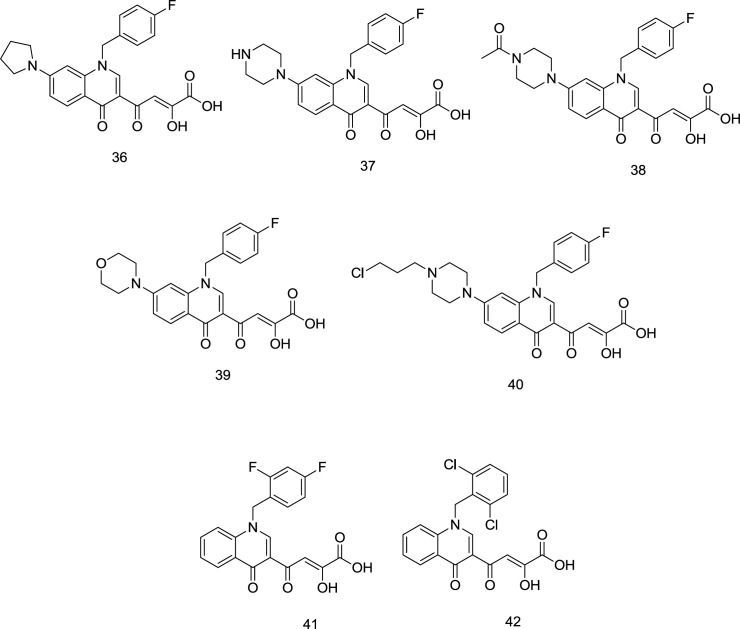
Quinolinonyl DKA derivatives

The high anti-HIV activity exhibited by the quinolinonyl diketo acid derivatives with a basic functional group like 1-pyrrolidinyl on position 7, exemplified by the compound **36** (RNase H IC_50_ = 28 nM and IN IC_50_ = 5.1 μM), compelled scientists to synthesize more ligands of this family (compound **37**, Figure 15) as RT-associated RNase H/IN dual inhibitors (97). Like pyrrolyl DKA analogs, carboxylic acid-containing compounds were more potent INST inhibitors than their corresponding ester homologs. 

However, in the cell-based antiviral study, the ester homologs were more potent, indicating lower cell membrane penetration by the acid derivatives (98). Among the newly-synthesized quinolonyl diketo acid derivatives, compounds **38 **(RNase H IC_50_ = 3.3 μM and INST IC_50_ = 80 nM), **39 **(RNase H IC_50_ = 6.8 μM and INST IC_50_ = 80 nM), and **40 **(RNase H IC_50_ = 5.7 μM and INST IC_50_ = 50 nM) (Figure 15) were identified as the most potent inhibitors for both of the enzymes. Interestingly, the least-active acid-containing INST inhibitor was compound **37** (IC_50_ = 2.0 μM), which possessed an unsubstituted piperazine moiety at C_7_. Further SAR studies on quinolinonyl DKAs indicated that quinolonyl N_1_-substitution with 2, 4-difluoro benzyl motif in compound **41** (Figure 15) leads to a high degree of IN inhibitory activity. However, 2, 6-dichloro benzyl in compound **42** was more favorable for RNase H inhibitory activity.

**HEPTs**

*HEPT analogs*

The first NNRTI candidate to be introduced was 1-[(2-hydroxyethoxy)-methyl]-6- (thiophenyl)-thymine (HEPT) (99) (Figure 12). Many HEPT analogs have been developed as candidates for clinical trials by then. According to the X-ray crystallography analysis data, all NNRTIs assume a two-winged conformation in HIV-1 NNRTI binding pocket (NNRTIBP) and show similar binding patterns (100). The HEPT analogs interact with the NNRTIBP via a strong hydrogen bond formed between their 3-NH and the carbonyl oxygen of Lys101, in such a way that the mutation of Lys101 leads to a considerable decrease in the efficiency of HEPT type NNRTIs (101). 

The SAR analysis showed that the interactions of HEPT C_6_-benzyl with Tyr181 had been significantly influenced by C_5_ alkyl (101-102). The 3,5-dimethyl group on the C_6_ benzene ring was notably in favor of RT binding, providing additional van der Waals interactions with the roof of the binding pocket. A carbonyl group at the C_6_ position usually leads to potent RT/IN dual activities and less cytotoxicity. The exchange of C_5_ and C_6 _position substituents would lead to a decreased activity, and compounds with a C_5_/C_6 _fused benzene ring were devoid of IN inhibitory activities. 

Furthermore, it was found that the N_1_-substituent anchors into a hydrophobic cavity made up of Val106, Pro225, Pro236, and Phe227 residues, and a wide variety of substituents are permitted to be placed at N_1_ position to improve binding affinity (101). 

**Figure 16:**
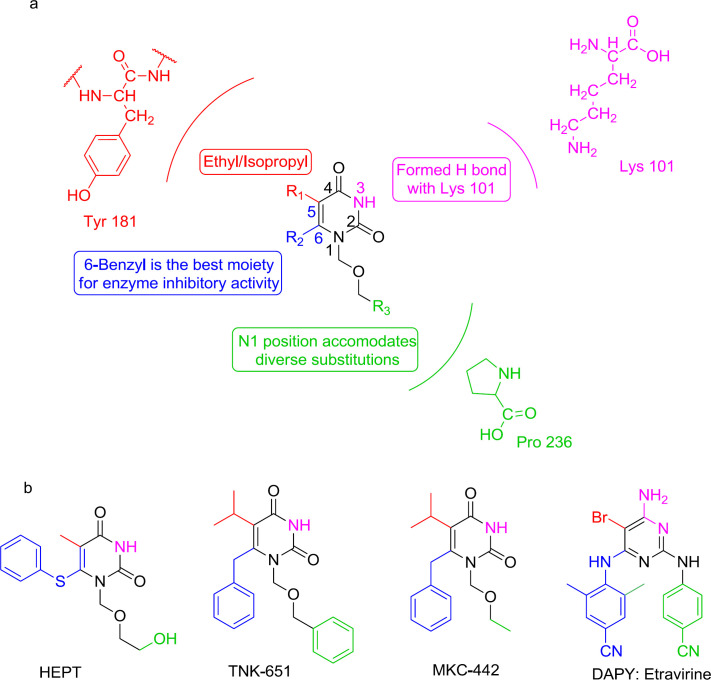
(a) Interactions between HEPT and the NNRTI pocket; (b) HEPT derivatives

Interestingly, the HEPT structure extends from the NNRTI pocket toward the aqueous solution and has an affinity to integrate a DKA mode (103). It was found that N_1_ linker substitution with a benzyl group affects dual RT/IN inhibitory activity in a manner that was dependent on the nature of C_5_ and C_6_ substituents (104). The inhibitors carrying an N_1_ side chain without benzyl group showed a complete loss of activity against IN, demonstrating that N_1 _substituting benzyl was vital to bind with IN (105). The N_1_-benzyl group in the side-chain was more favorable than the C_6_ benzyl group for inhibition of IN activities (106). Halogenation at the para position of the N_1_ side-chain benzyl group was observed to be beneficial to increase IN binding affinity. The optimal IN inhibition required a linker of 3-4 atoms (107); lengthening of the N_1_ linker adversely affected IN binding affinity. This knowledge of SAR paved the way for discovering a novel diaryl pyridine class of drugs like etravirine (Figures 8 and 16).

*N*_3_ *-Hydroxy HEPT analogs*

**Figure 17:**
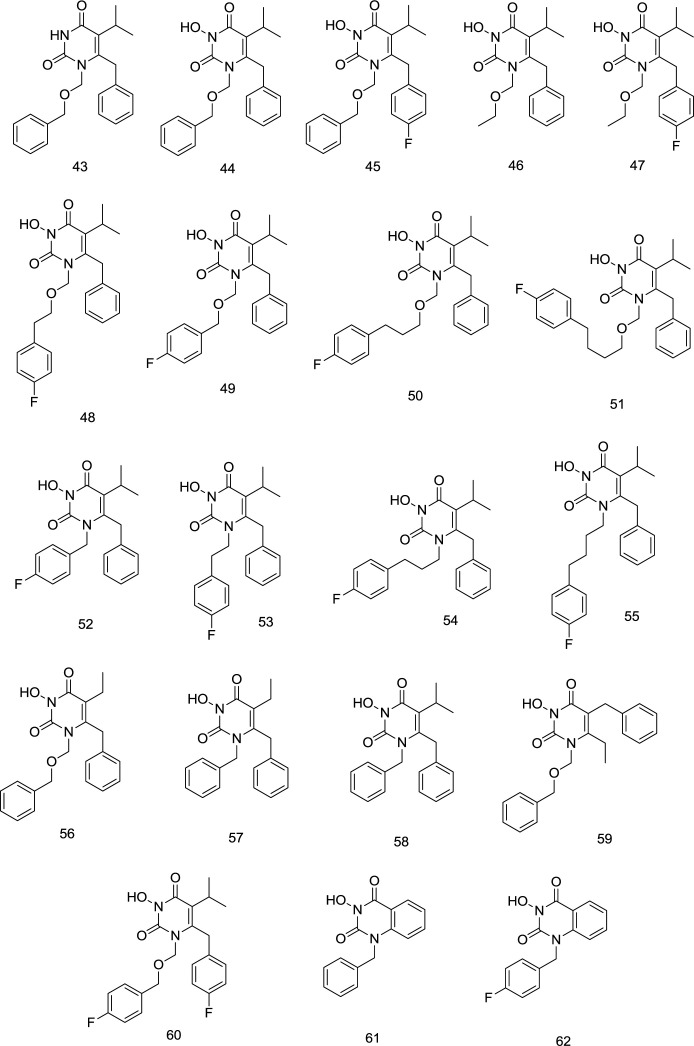
N-3 Hydroxy HEPT analogs

In their next study, Wang *et al.* created N_3_-hydroxylated HEPT analogs **44-47 **(Figure 17)**,** considering that OH could form a hydrogen bond with one of the four successive Lys101-Lys104 residues of NNRTI binding pocket. It was noteworthy that the parent compound **43** did not exhibit any inhibitory activity against IN, suggesting that the N_3_-hydroxylation is necessary for metal coordination. By contrast, N_3_-amination produced inactive compounds inferring that the N_3_-hydroxy motif offers a better chelating triad. Compounds **46** and **47** demonstrated considerably reduced INST inhibition compared to compounds **44** and **45**, signifying that the terminal benzyl group at the N_1_ chain is much more essential for IN binding than the one on pyrimidine-2,4-dione C_6_ (106). It was found that the halogenation of N_1_-benzyl significantly enhanced IN binding efficiency. A six-time enhancement of IN inhibition activity was reported from para-fluorinated compound **45** (IC_50 _= 3.5 μM), compared with the un-substituted homolog **44** (IC_50 _= 20 μM). In short, the two main elements required for the IN inhibition include N_3_ hydroxylation and benzyl para-fluoro substituents. Vince and Wang reported analogs of **43** containing a benzoyl group at C_6_ position central of pyrimidine ring, which inhibited RT, IN, and HIV-1 at nM to low μM range. The findings were in accordance with the molecular modeling studies, in which the chelation of Mg^2+^ was done by the “OC-N (OH)-CO” key component that replicates a DKA pharmacophore (107-108). INSTI activity is found to be influenced by the longitude and the essence of the linker domain. Computational studies indicate the linker domain between DNA nucleobases & Pro145 in hydrophobic pockets. Among the connections, the most inhibitory effect was observed with - (CH_2_)_2_OCH_2_- (**48**). By contrast, a reduction was seen in compounds with a small (**49**) or long linker (**50-51**). The shorter all-carbon connections (**52** and **53**) appear to pose binding difficulties, but with three and four atom connectors (**54** and **55**), INSTI inhibition has been greatly improved. Both carbon-connecting inhibitors demonstrated considerably better than compounds of a comparable length ether linkage (**55** *vs*. **48**).

Nonetheless, in cell-based experiments against HIV, all-carbon binders were found to be ineffective due to adverse physicochemical properties. The compounds carrying (-CH_2_OCH_2_-) linkers were then chosen for further development because of their mM IN inhibition and nM anti-HIV action. The effect of steric changes to the hydrophobic domain resulted in the changes to the hydrophobic profile of the molecule and presented a mixed picture. Still, it seems to be sterically influenced by aromatic and the N_1_ linker domains. In this respect, the size of C_5_ and the halogenation of C_6_ benzyl were of considerable importance. At C_5_, it was seen that less bulky ethyl (**56**) was superior to isopropyl (**43**) for INSTI as compared to RTI.

On the contrary, in compounds possessing an alkyl, only N_1_ linker of CH_2_ and ethyl at C_5_ (**57**) caused a complete loss of IN inhibitory activity compared to C_5_ isopropyl substitution (**58**) with the same methylene linker. Swapping C_5_ and C_6_ substituents (**59**) seemed to harm IN binding, whereas a para-fluoro substitution at the C_6_ benzyl (**60**) favorably influences IN inhibition (**44** *vs*. **59**). Compounds **61** and **62** were utterly inactive to inhibit IN activity in the case of an infused benzene ring between C_5_ and C_6_.

*C*_6_ *modification of N*_3_ *- hydroxyl HEPT analogs*

**Figure 18:**
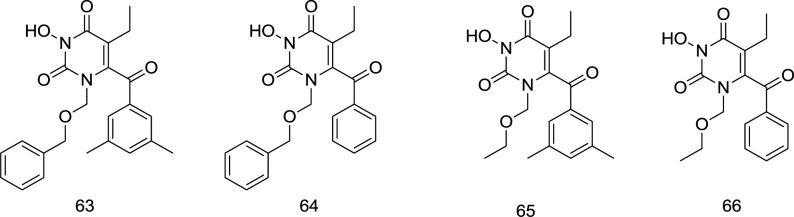
*C*-6 modification of HEPT analogs

In 2011, an extended series of N_3_-hydroxylated pyrimidine-2, 4-diones featuring a benzoyl group at the C_6_ position of the pyrimidine ring was introduced by Tang *et al.* as dual HIV-1 RT/IN inhibitors in the low micromolar range (108). The compound **63** (Figure 18) substituted at C_6 _position with the 3, 5-dimethyl benzoyl motif appeared to be a potent inhibitor of both the HIV-1 RT and IN enzymes, where the dimethyl group on the C_6_ benzene provides additional van der Waals interactions with the RT binding pocket. On the contrary, fluorination of the N_1_-benzene and the extra carbon in the N_1_ linker were found to confer a substantial decrease in RT inhibition. Intriguingly, a reverse trend was observed in IN inhibition, in which placing the fluorine or adding extra carbon to the N_1_ linker was shown to slightly favor IN inhibition. 

*5-N-benzylcarboxamide modification of N*_3_ *-hydroxy HEPT analogs*

The Wang group’s persistence with the promising 3- hydroxypyrimidine-2,4-dione (HPD) **44** inhibitor has culminated in one of the strongest HIV-1 RT related RNase H inhibitors contrasted with the fact that the assigned nucleus also seems to have outstanding INST inhibition properties. N-benzyl-5-carboxamide moiety created **67** from **44,** possessing a similar structure to dolutegravir (**18**). SAR studies stressed the significance of an aminoalkyl group at C_6_ and an N-benzyl group on the C_5_ carboxamide for INST inhibition and antiviral activity. Interestingly, studies in molecular modeling and docking showed that **67** binds to the IN site in a dolutegravir-like mode. Antiviral assessment of **67** against HIV-1 strains resistant to raltegravir showed the same resistance profile as dolutegravir. Moreover, the RNase H activity was strongly affected by similar chemotypes, suggesting that dual RT/IN inhibition may be the primary mechanism of the antiviral activity. The ADME analysis also verified the drug-like physicochemical properties of this new chemotype.

**Figure 19:**
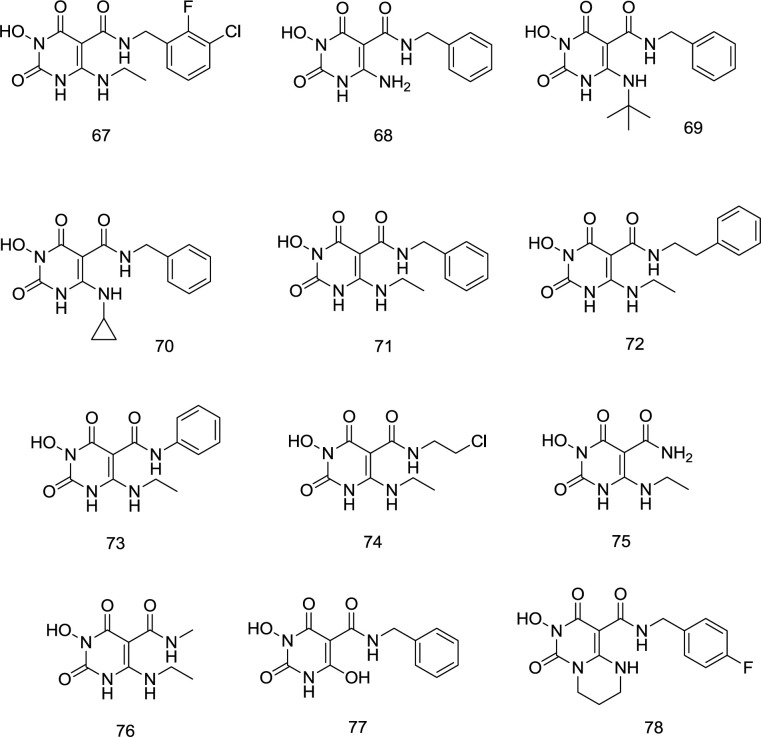
5-N-benzylcaboxamide modification of N_3_-hydroxy HEPT analogs

Most importantly, analogs substituted at C_6_ position by aromatic amine were entirely ineffective for INSTI and mostly cytotoxic, whereas small aliphatic amines exhibit impressive antiviral activity without cytotoxicity. Even though most aminoalkyl analogs provided maximum INSTI activity without significant cytotoxicity, linear aminoalkyl analogs such as **67** were mainly preferred in the INSTI assay, compared with unsubstituted **68**, bulky alkyl **69**, and cyclopropyl **70**. The best INSTI activity was reported for the aminoethyl substituted **67 **(Figure 19).

Concerning the C_5_ carboxamide group SAR, a novel series of compound **71** derivatives was synthesized and assessed as INSTIs. Curiously, all products with a benzyl moiety on the C_5_ carboxamide have provided maximum cytoprotection at a concentration of 10 mM without considerable toxicity. Exceptional IN inhibition with IC_50_ values of 21 to 230 nM was also observed. Interestingly, compounds with extra methylene (**72**) have developed high cytoprotection, although IN inhibition has decreased significantly. Antiviral activity was killed either by the benzyl switching with a phenyl (**73**) or an alkyl group (**74**) or by the elimination of an N-substituent (**75**) (Figure 19). Antiviral function, cell viability, and IN enzyme inhibition activity are eliminated by N_1_ methylation (**76**). C_5_ carboxamide methylation also resulted in a total loss of antiviral activity, while biochemical inhibition was significantly reduced. The antiviral and IN inhibition activity of 6-deamino derivatives (**77**) have been considerably reduced in the same way. Cyclic analog **78** was both inactive and cytotoxic during the antiviral examination. However, enzyme inhibition remained essentially unchanged.

**Pyridopyrazine analogs**

**Figure 20:**
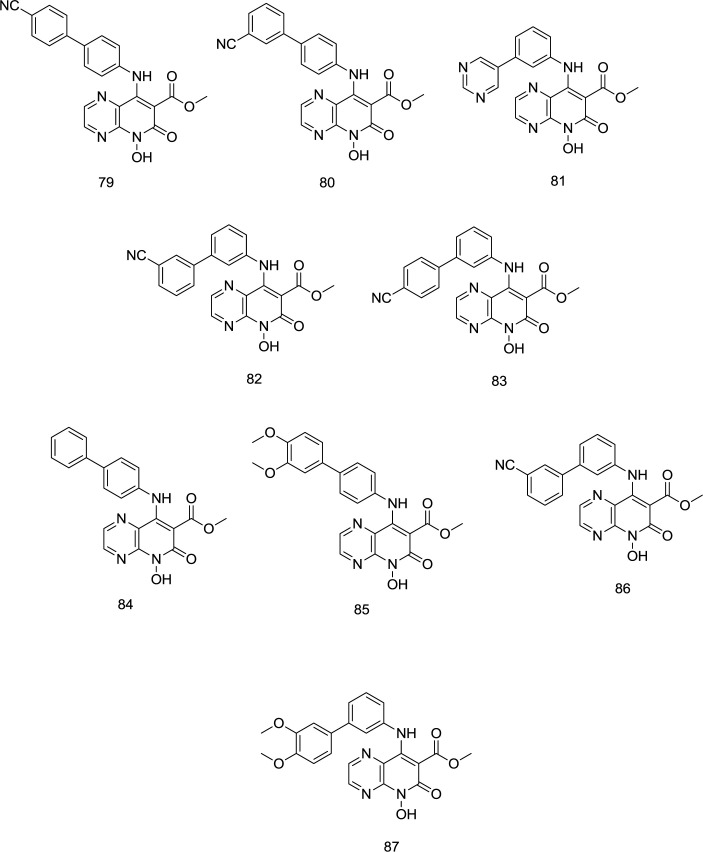
Pyridopyrazine analogs

Using the analog-based optimization of divalent metal-chelating motif, a novel series of pyrido[2,3-b]pyrazin-6 (5H)-one analog bearing a hydroxy group on C_2_ position were developed by Sun and coworkers as dual-acting HIV-1 RNase H/IN inhibitors (109), in which the para-substituted benzene analog **79** (Figure 20) proved to exhibit similar inhibitory activity against RNase H and IN with IC_50_ values of 1.77 and 1.18 µM, respectively. Compared to para-substituted phenyl, the meta-substituted analogs were much more selective against RNase H. However, as an exception, the replacement of 4-cyanophenyl by 3-cyanophenyl in compound **80** was found to cause a reduction in dual RNase H/IN inhibition activity. Oppositely, in meta-substituted benzene analogs, compounds **81** and **82** (Figure 20), bearing 5-pyrimidyl and 3-cyanophenyl moieties, respectively, were more effective than compound **83** with a 4-cyanophenyl. Interestingly, all the compounds possessed a low IC_50_ RNase H/IC_50 _IN ratio, suggesting that they have an appropriate balance in inhibiting RNase H and IN.

Substitution with the electron-donating methoxy, exemplified by the **85** and **87**, indicated negligible effect against RNase H activity. Compounds **86** and **87** were the most potent IN inhibitors with IC_50_ values of 0.25 and 0.22 mM, respectively. Similar to the RNase H, meta-substituted benzene analogs showed better IN inhibitory activity than the para-substituted benzene derivatives (exemplified by **86** and **87**). Furthermore, the para-CN phenyl group produced more potent drugs among the para-substituted phenyl analogs than meta-CN phenyl substitution, while the pattern for meta-substituting phenyl derivatives was contrary. Compound **79** supplied a balanced RNase H and IN micromolar inhibition, validating the hypothesis that correctly configured hydrophobic and chelating pharmacophores may provide a viable RNase H/IN dual inhibitor.

**Quinazoline analogs**

**Figure 21:**
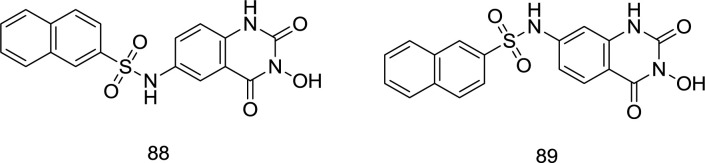
Quinazoline analogs

Gao *et al.* worked with 3-hydroxyquinazoline-2, 4 (1H, 3H)-diones and prepared various derivatives as dual inhibitors of HIV-1 RT-associated RNase H and IN (110) (Figure 21). The newly-synthesized analogs were yielded by hybridizing hydroxyl isoquinoline (HID) and hydroxypyrimidine-2, 4-dione (HPD) scaffolds. In the biological assay, the benzenesulfonyl analog **88,** which was substituted with a naphthyl group at the C_6_ position, acts as the best with an IC_50_ of 0.41 μM that was nearly five times lower than that of the IC_50_ with β-thujaplicinol. Furthermore, it was found to be effective in inhibiting HIV-1 IN strand transfer activity with an IC_50_ value of 0.85 μM. 

It was found that the shifting of the naphthyl group from C_6_ in compound **88** to C_7_ in **89** leads to a fifty times reduction of the RNase H inhibitory strength, which is found to diminish in the following order: naphthyl > phenyl > 3-CF_3_ phenyl > 4-F phenyl. Interestingly, the pattern of changes in the RNase H and IN inhibitory power of some compounds was similar. 

**Isoquinoline analogs**

**Figure 22:**
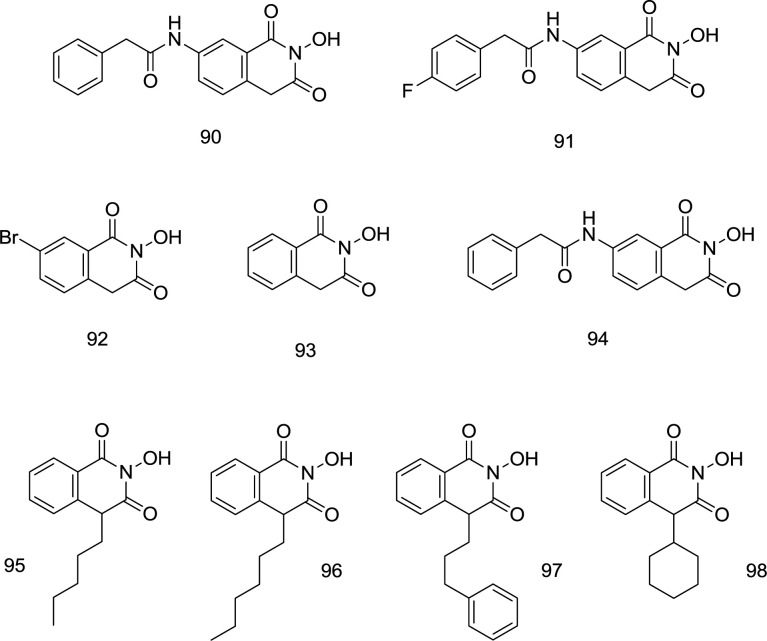
Isoquinoline analogs

In 2008, two novel hits **90,** and **91** (Figure 22) were discovered by Billamboz *et al.* through the synthesis of variously C_7_-substituted 2-hydroxyisoquinoline analogs which displayed promising selective integrase inhibitory activity with submicromolar IC_50_ values (0.09 μM for **92** and 0.13 μM for **93**) along with a moderate effect against RNase H at a micromolar concentration (111). The metal coordination was shown to be strictly dependent on the enolizability of the ligands. For the HIV-1 IN specificity, the structure prerequisite may seem to be a phenyl acetamide group on C_7_ that makes it possible for classical HIV-1 IN inhibitors to fit well into the pharmacophore model. 

A high degree of selectivity for the strand transition was also inducted through fitting the hydrophobic aromatic zone, to which the low selectivity of **92** compared to the high ST phase specificity of **90** and **91** attests (Figure 22). Through working on the hydroxyisoquinoline-1,3- (2H,4H)-diones (**93**), Billamboz’s research group developed several C_7_ substituted derivatives (like **94, **Figure 22). Polymerase inhibition was not observed; however, metal coordination-based RT RNase H and IN inhibitory activities were observed with a high degree of selectivity towards IN. All compounds were found to be highly cytotoxic. The degree of the enolation relied on the metal chelation. The C_7_ substituted analogs displayed INI activity potentiation but did not improve the dual RNase/IN inhibition (112). The studies were followed up by modification of C_4_ (113). In most 4-substituted derivatives, RNase H inhibitory activity was eliminated; however, two compounds (**95** and **97, **Figure 22) exhibited encouraging IN inhibition activity, with a considerable enhancement in IC_50_ values than parent structure **92**. Substitution of alkyl or alkyl aryl chain at C_4_ led to moderate IN inhibitors with micro-molar activities (**95** and **97**) with a 2-4.5-fold increase in integrase inhibition, in comparison to the unsubstituted compound **93**. The high integrase inhibitory activity of **95** and **97** was compatible with the docking findings. The improvement of the INI was due to the hydrophobic interactions with terminal 3’-adenosine of virus DNA at the IN site, and both simultaneous enzyme- and viral DNA interactions were expected to occur in the **97**-phenyl propyl group. The Mg^2+^ coordination and the accompanying interactions between hydrophobic zones in the IN active site are still not adequate for these molecules to induce a considerable enzyme inhibition. Even with C_4_ alkylation, a new class of anti-HIV drugs was incomplete. However, the optimization of this quinoline scaffold still holds a promising drug candidate’s potential.

**Chromenone/Coumarin-based analogs**

**Figure 23:**
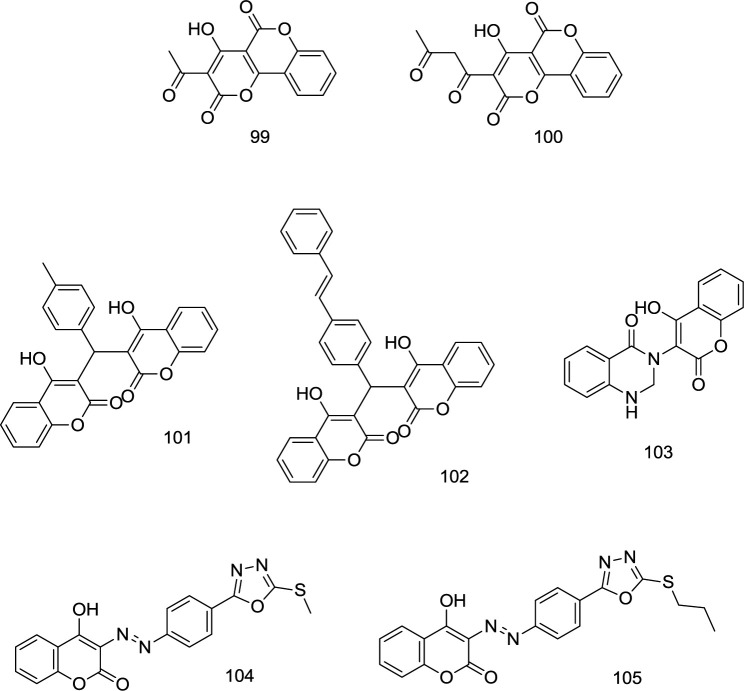
Chromenone/Coumarin-based analogs

In a study by Esposito and coworkers, several versatile coumarin-based analogs with dual mode of inhibition of RNase H/IN were developed, in which compound **99 **(Figure 23) proved to possess high antiretroviral potency with IC_50_ values of 6.25 and 6.45 μM for RNase H and IN, respectively (114). Some coumarin-based analogs with promising antiretroviral activities were introduced as novel IN inhibitors in our previous works (27, 29 and 115-117) (compounds **100-105**); however, their RNase H inhibitory effects were remained to be investigated. 

**Hybridized analogs**

*DKAs-delavirdine hybrid analogs*

**Figure 24:**
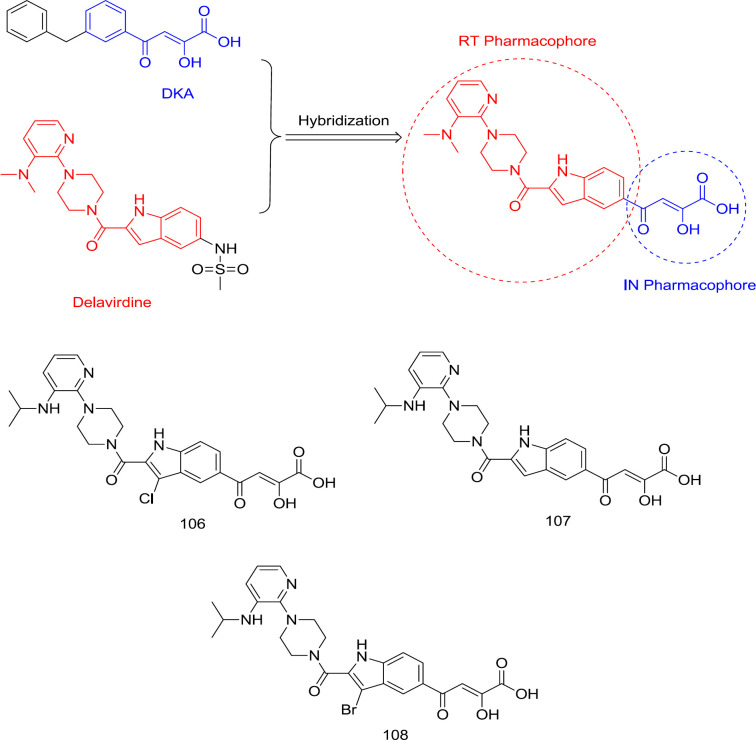
Hybridization of delavirdine and DKAs as dual RT/IN inhibitors

In a study led by Wang, a DKA motif was substituted with the C_5_ methyl-sulfonamide group of an FDA-approved RT inhibitor, delavirdine, to produce a new RT/IN group dual inhibitors with sub- or low micromolar activity against RT and IN in enzymatic assay (118) (Figure 24). A balanced binding affinity against RT and IN was achieved by compound **106** (Figure 24) with IC_50_ of 1.1 and 4.7 μM against RT and IN, respectively. This family of compounds seems to take on a pseudo-linear spatial conformation, which benefits both RT and IN binding. On the contrary, compounds with DKA substitution at the C_7_ position were virtually inactive against both enzymes. Notably, the indole C3 site’s substitution with a halogen was also observed to reduce the RT inhibitory activity while slightly improving the anti-IN potency. 

SAR analysis showed that the hybrid ligands possessing DKA pendant on the C_5_ position, including compounds **107 **(RT IC_50_ = 5.9 nM and IN IC_50_ = 12 μM) and **108 **(RT IC_50_ = 120 nM and IN IC_50_ = 3.9 μM) (Figure 24) took a pseudo-linear conformation like delavirdine, which benefits both IN and RT binding. It was also found that substitution of indole’s C_3_ position with bromine leads to a lower anti-RT and higher anti-IN activity, thus reducing the disparity observed between both activities (119). Therefore, it was found that specific RT inhibitor scaffolds and resulting dual inhibitory properties can be introduced into IN central DKA pharmacophore without substantial loss of the RT inhibition.

*DKAs-HEPT analogs*

**Figure 25.**
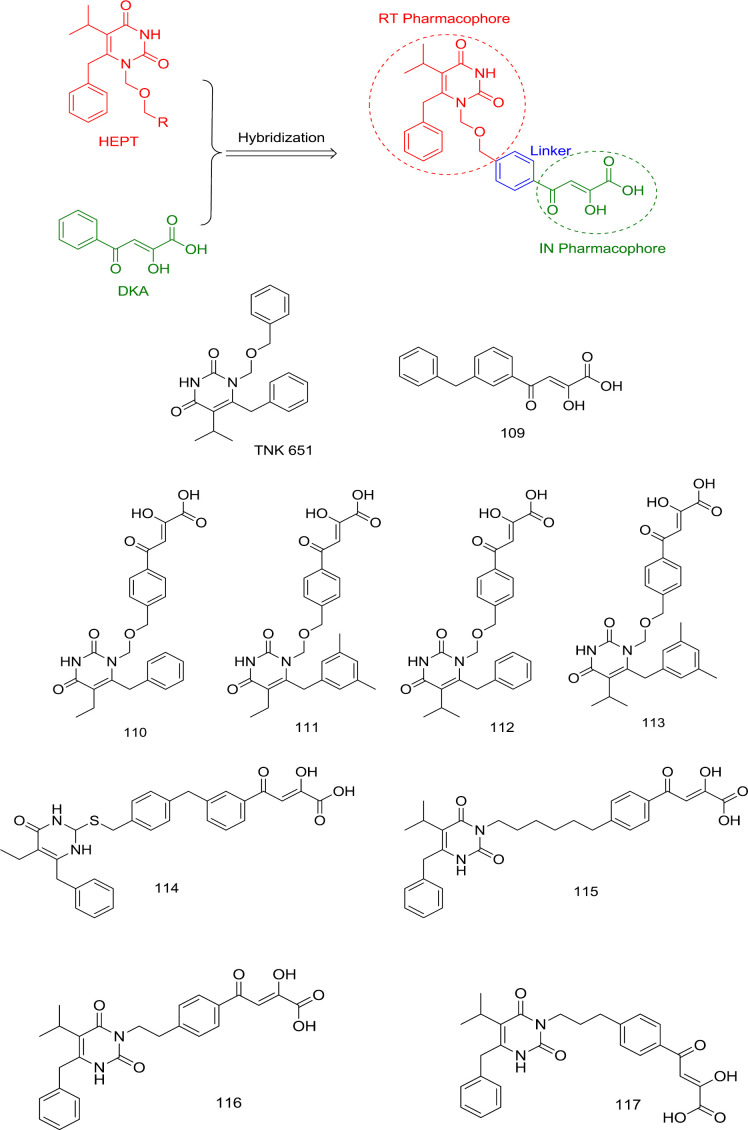
Hybridization of HEPTs and DKAs as dual RT/IN inhibitors

In 2007, Wang *et al.* exploited HEPT N_1_ benzyl’s proximity to the Pro236 at the NNRTIBP to develop a novel generation of RNase H/IN dual inhibitors. They combined an IN inhibitor (**109**) DKA moiety with HEPT-derived TNK 651 at its N_1_ benzyl terminus to produce compounds **110-114** (Figure 25) without any adverse effects on the intrinsic inhibitory activity of both the combined moieties (103). 

Based on these studies’ findings, it was identified that substitution of N_1_ with another benzyl side chain or lengthening the N_1_ linker hampers IN binding, suggesting that only the C_6_ benzyl group fulfills the hydrophobic pharmacophore requirement for IN inhibition. Further SAR studies established that the shifting of DKA moiety to the C_2 _positions of the HEMP pyrimidine nucleus caused considerable loss of IN inhibitory activity, as observed in compounds **114** and **115** with IN IC_50_ > 100 μM. Moreover, to obtain an optimal RT/IN dual inhibition in HEPT derivatives, the linker between DKA and HEPT ring should be 2-3 atoms long, as observed in compounds **116** and **117** (104).

*DKAs-DAPY analogs*

Gu and coworkers fused the classic IN inhibitor pharmacophore of diketo acid with the left-wing of the DAPY type NNRTI.

**Figure 26:**
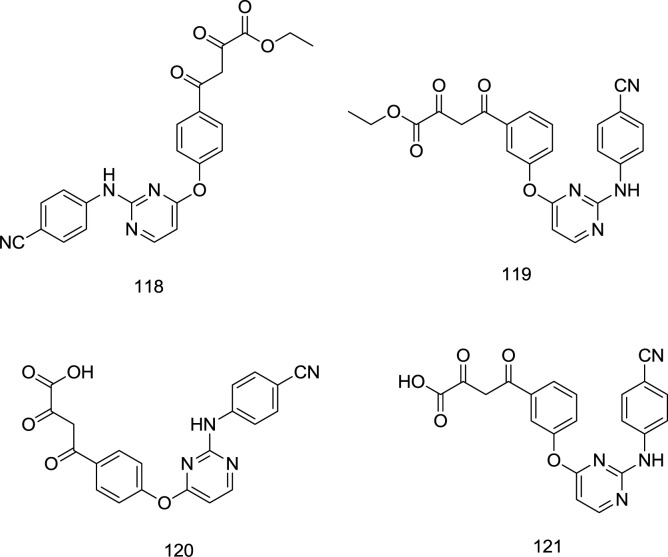
DKAs-DAPY analogs

Keeping the crucial right wing intact, they reported significant NNRTI activity retention and docking pose alignment between the derivative and the parent NNRTI (120) (Figure 26). The best antiretroviral activity was reported from the analog **120** with an HIV-1 EC_50_ of 0.23 μM. 

*HEPT-quinolone hybrid analogs*

**Figure 27:**
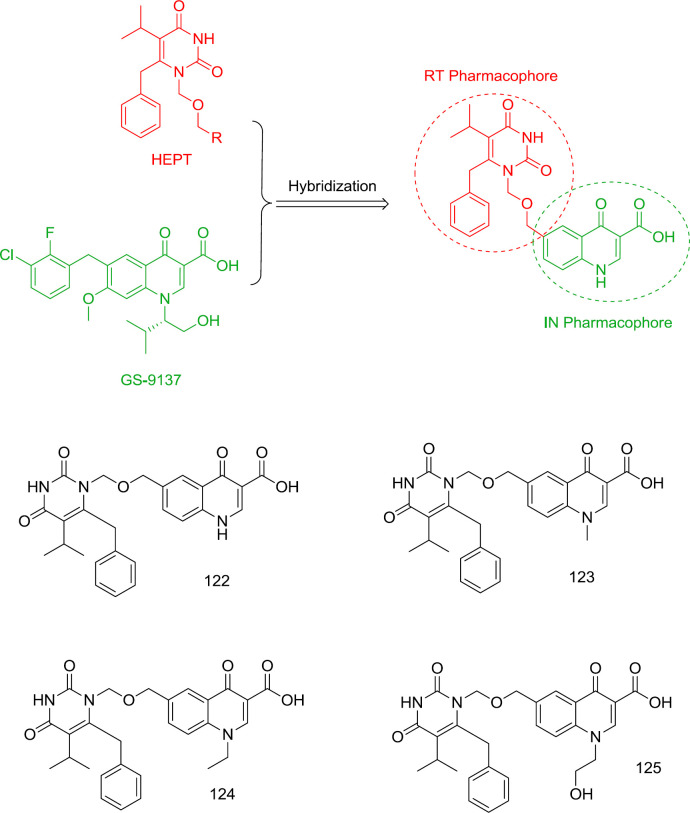
HEPT-quinolone hybrid analogs

One of the oldest attempts to design dual RT/IN inhibitors with inhibitory activity at low to sub-micromolar levels was carried out by Wang and Venice in 2008 by combining pyrimidine and GS-9137 quinolone moiety as an oral HIV integrase inhibitor (121) (Figure 27). It was found that incorporation of quinolone carboxylic acid at the N_1_ terminus of HEPT leads to the potential dual RT/IN inhibitors **122-125**, among them, the most RT and IN inhibitory activities were observed from compounds **122 **(RT IC_50_ = 0.19 μM, IN IC_50_ = 35 μM) and **125 **(RT IC_50_ = 3.7 μM, IN IC_50_ = 19 μM), respectively. 

*Pyridine-AZT and Coumarin-AZT hybrid analogs*

**Figure 28:**
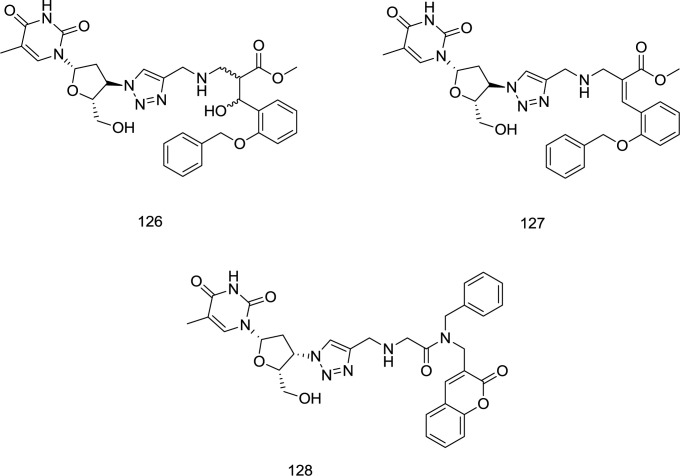
AZT hybrid analogs as dual RT/IN inhibitors

Employing Baylis-Hillman methodology, a novel series of 3-hydroxy-3-phenylpropanoate ester-AZT conjugates has been prepared by Manyeruke *et al.*, as well as their potential as dual inhibitors of HIV-1 IN/RT inhibitors has been explored using nevirapine and raltegravir, as standards for the RT and IN assays, respectively (122). Among the newly-synthesized analogs, compounds **126** and **127** were identified as the most encouraging agents (Figure 28).

*Pyrimidine-raltegravir hybrid analogs*

**Figure 29:**
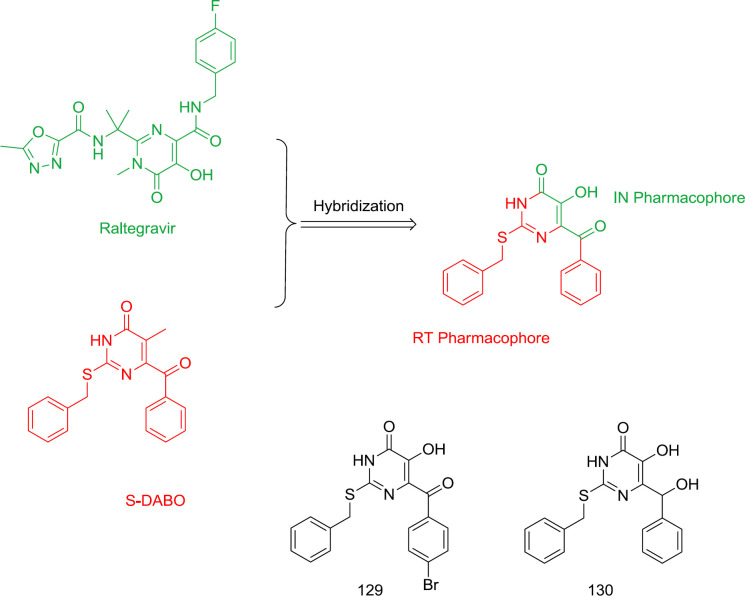
Raltegravir-DABO hybrid analogs

Dihydroalkyloxybenzyloxopyrimidines (DABOs) were identified as potent HIV-1 NNRTIs (123). Replacement of DABOs C_2_ oxygen with sulfur generated a new series of S-DABOs with potent activity and less toxicity. Hydroxyl group substitution at the C_5_ in S-DABO created a hybrid of raltegravir and S-DABO as dual RT/IN inhibitors, as are shown in Figure 29. Unfortunately, the anti-RT and antiretroviral activities of the newly-synthesized hybrid analogs were reduced to low levels, in which compounds **129** and **130** were found to be inactive against RT.

*Pyrimidine-quinolone hybrid analogs*

**Figure 30:**
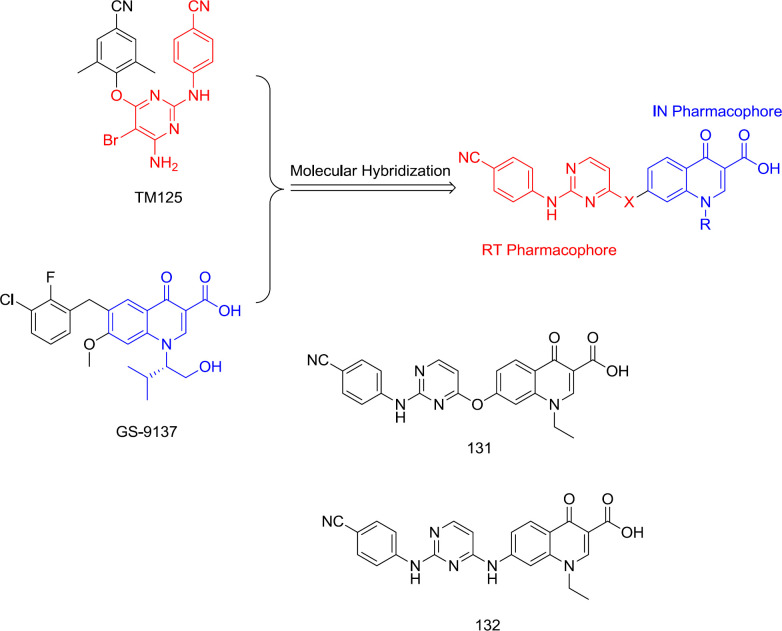
Pyrimidine-quinolone hybrid analogs

In 2015, TM125 (RT inhibitor) and GS-9137 (IN inhibitor) were hybridized into a series of diarlyprimidine-quinolone hybrids (Figure 30) as RT/IN dual inhibitors, exemplified by the analogs **131** and **132**. However, all the compounds were devoid of IN activity at very high concentrations, demonstrating that polar quinolone-3-carboxylic acid moiety is unsuitable for replacing the hydrophobic aryl wing of classical DAPY (124). 

**Natural-derived analogs**

**Figure 31:**
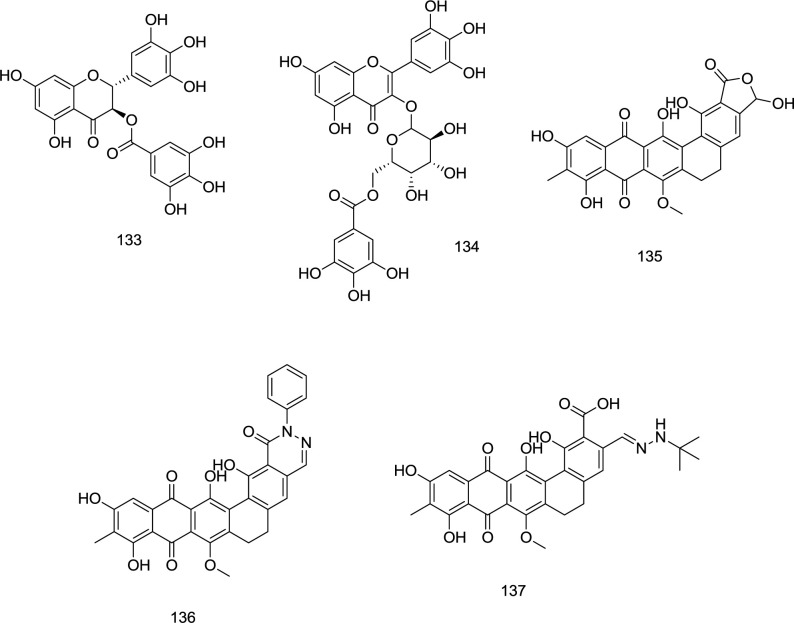
Nature-derived RT/IN dual inhibitors

During a search for potential templates of HIV-1 RT/IN dual inhibitors, the methanolic extract obtained from *Limonium morisianum* was investigated by Sanna *et al.* (125). Among the isolated molecules, (-)-epigallocatechin-3-*O*-gallate **133** and myricetin-3-*O*- (6″-*O*-galloyl)-β-d-galactopyranoside **134** potently inhibited both enzymes with IC_50_ values ranging from 0.21 to 10.9 μM (Figure 31).

Madurahydroxylactone **135** (Figure 31), a metabolite from the soil bacterium *Nonomuraea rubra*, belongs to the naphthacenequinone antibiotics family. In a study by Marchand *et al.*, a novel series of madurahydroxylactone analogs, exemplified by the analogs **136** and **137** (Figure 31), was developed as dual RNase/IN inhibitors in which most compounds exhibited high inhibitory activity against both enzymes.

**Expert Opinion**

It is a widespread opinion that DMLs could offer greater therapeutic efficacy and less drug resistance relative to the single-target medications. Nevertheless, despite substantial work on DMLs and development, there is a lack of a success story, and most compounds are powerless relative to their parent compounds. It is essential to recognize that a balance must be made in terms of the specificity of a structure when designing multiple ligands, which leads to a loss of power compared with the parent compound within the DML. Researchers were able to produce these DMLs and maintain the individual inhibitors’ pharmacophore properties during the active inhibitors’ association. However, the individual inhibitors’ flexibility helped them bind to the target receptive site via adopting the appropriate conformation has not been preserved. DMLs for IN, RT, and the RNase H active sites were extensively worked out, achieved encouraging results. However, how to improve the potency and simultaneously balance the RT/RNase H inhibitory activity is still a challenge. Currently, there is not enough data to derive a logical, comprehensive SAR for dual RNase H/IN inhibitors. We know now that all of these compounds have a metal coordinating motif and a lipophilic side group. These two pharmacophores are necessary to bind to both enzymes’ active site, but the way they are placed together makes one a more potent inhibitor against IN, while the other acts as a specific RNase inhibitor. Based on current knowledge, it can be said that compounds with an ionizable acidic moiety show a greater tendency to bind to IN. Conversely, with non-ionizable chemical moieties like ester and amide, the RNase H inhibitory effect is found to be more significant. Moreover, it was discovered the closer the angle between the metal-coordinating group and the hydrophobic ring to 120°, the higher the IN inhibitory effect. Clearly, this is by no means sufficient to design ligands that inhibit both IN and RNase H enzymes in a balanced manner. Especially we still do not know precisely where the lipophilic group’s best position. More docking studies and quantitative structure-activity relationship (QSAR) analysis should be performed to understand the best way to configure these two groups, and more diverse ligands should be designed and evaluated. A general disadvantage of RNase H is that it has a human homolog, which makes it somewhat challenging to design dual inhibitors that specifically inhibit the viral enzyme and keep the human homolog intact. As a result, it may be helpful to find biological targets other than IN that can be inhibited simultaneously with IN. Otherwise, new techniques for exploiting IN inhibition are under-worked through inhibiting viral DNA-IN attachment or preventing the incorporation of lens epithelium-derived growth factor (LEDGF)/p75, an essential HIV-1 integration cofactor (126-128). Go for more goals, like LEDGF/p75, CXCR4 (129) or CCR5 (130) can result in additional penalization of potency and selectivity. Nevertheless, one should not be disappointed with the RNase H inhibitors as promising antiretroviral agents. Unlike the human type of RNases H, the retroviral homolog possesses three modes of cleavage: internal, DNA 3′ end-directed, and RNA 5′ end-directed, all of them appear to play crucial roles in reverse transcription (131). Relevant RNase H inhibitors could be identified using the details on the nature of these interactions.
